# Supplementary material for: The impact of different data handling strategies on the proportion of children classified as meeting 24-h movement guidelines and associations with overweight and obesity
Source: J Act Sedentary Sleep Behav. 2024 Jan 2;3:1. doi: 10.1186/s44167-023-00041-5 (PMC11960250; doi:10.1186/s44167-023-00041-5)
Supplement: Supplementary file 1 — Additional file 1. Supplementary Table 1. Summary of the impact of data handling strategies on estimates of meeting 24hrG (N = 524). Supplementary Table 2. Summary of the impact of data handling strategies on the associations with meeting guidelines and odds of overweight/obesity (OWOB) (N = 475). [file 44167_2023_41_MOESM1_ESM.docx]

| Data Handling Strategy | Description of Strategy | Movement Behaviors | | | |
| --- | --- | --- | --- | --- | --- |
|  |  | MVPA | Sleep | Screentime | All Three |
|  |  | Prevalence Estimates | | | |
| AVG-24hr | Daily estimates were averaged.  Averages were dichotomized into “met guidelines” and “did not meet guidelines” for the total sample | 59.4% met guideline | 54.9% met guideline | 33.2% met guideline | 14.7% met guideline |
| DAYS-24hr | Each individual day was dichotomized as “met” or “did not meet” a guideline | 8.8% met guideline on 100% of measured days | 5.7% met guideline on 100% of measured days | 6.8% met guideline on 100% of measured days | 0.2% met guideline on 100% of measured days |
| RAND-24hr | Random sampling of four days was repeated over 10 rounds and the total number of times participants met guidelines using those four days of data was calculated for each round of randomization | The range of percent meeting guideline across 10 rounds was 54.9-57.5% | The range of percent meeting guideline across 10 rounds was 54.0-57.5% | The range of percent meeting guideline across 10 rounds was 36.4-39.0% | The range of percent meeting guideline across 10 rounds was 15.8-16.8% |

**Supplementary Table 1**. Summary of the impact of data handling strategies on estimates of meeting 24hrG (N=524).

**Supplementary Table 2.** Summary of the impact of data handling strategies on the associations with meeting guidelines and odds of overweight/obesity (OWOB) (N=475).

| Data Handling Strategy | Description of Strategy | Movement Behaviors | | | |
| --- | --- | --- | --- | --- | --- |
|  |  | MVPA | Sleep | Screentime | All Three |
|  |  | Odds of OWOB (95%CI) | | | |
| AVG-24hr | Daily estimates were averaged.  Averages were dichotomized into “met guidelines” and “did not meet guidelines” for the total sample | 0.33  (0.21, 0.52) | 0.56  (0.36, 0.88) | 0.83  (0.55, 1.24) | 0.38  (0.21, 0.70) |
| DAYS-24hr | Each individual day was dichotomized as “met” or “did not meet” a guideline | 0.08  (0.04, 0.19) | 0.26  (0.10, 0.65) | 0.58  (0.30, 1.13) | 0.04  (0.01, 0.18) |
| RAND-24hr | Random sampling of four days was repeated over 10 rounds and the total number of times participants met guidelines using those four days of data was calculated for each round of randomization | 0.38  (0.25, 0.58) | 0.71  (0.47, 1.07) | 0.69  (0.46, 1.04) | 0.48  (0.27, 0.86) |
|  |  | 0.41  (0.27, 0.63) | 0.57  (0.37, 0.86) | 0.65  (0.43, 0.97) | 0.46  (0.26, 0.81) |
|  |  | 0.47  (0.30, 0.73) | 0.82  (0.54, 1.25) | 0.83  (0.55, 1.25) | 0.56  (0.33, 0.97) |
|  |  | 0.38  (0.24, 0.58) | 0.51  (0.33, 0.77) | 0.76  (0.50, 1.15) | 0.35  (0.19, 0.63) |
|  |  | 0.43  (0.28, 0.66) | 0.67  (0.44, 1.02) | 0.78  (0.52, 1.17) | 0.50  (0.28, 0.88) |
|  |  | 0.43  (0.28, 0.67) | 0.65  (0.43, 0.99) | 0.77  (0.51, 1.16) | 0.53  (0.30, 0.95) |
|  |  | 0.43  (0.28, 0.66) | 0.60  (0.39, 0.92) | 0.69  (0.46, 1.04) | 0.40  (0.23, 0.71) |
|  |  | 0.40  (0.26, 0.62) | 0.52  (0.34, 0.80) | 0.81  (0.53, 1.21) | 0.33  (0.18, 0.59) |
|  |  | 0.37  (0.24, 0.58) | 0.56  (0.36, 0.86) | 0.62  (0.42, 0.94) | 0.27  (0.15, 0.51) |
|  |  | 0.38  (0.25, 0.58) | 0.60  (0.39, 0.91) | 0.70  (0.47, 1.06) | 0.43  (0.24, 0.76) |
